# Financial and Psychosocial Consequences of COVID-19 for Dentists in Cyprus: Policy Dimensions

**DOI:** 10.7759/cureus.99030

**Published:** 2025-12-12

**Authors:** Chrystala Charalambous, Aristomenis I Syngelakis, Maria Myrto Solomou, Kostis Giannakopoulos

**Affiliations:** 1 School of Dentistry, European University Cyprus, Nicosia, CYP; 2 Oral Medicine, European University Cyprus, Nicosia, CYP

**Keywords:** covid-19, cyprus, dentistry, economic impact, financial crisis, pandemic, psychosocial impact

## Abstract

Background: In 2020, dentists were among the health professionals most affected by the COVID-19 pandemic. This study aimed to assess its financial impact on dentists in Cyprus during 2020-2021, compare it with the 2012-2013 economic crisis, and examine associated psychosocial effects.

Material and methods: An anonymous 54-item questionnaire was emailed to all 1,157 registered dentists in Cyprus, according to the official registry of the Cyprus Dental Association, between April and May 2022. To enhance the response rate, two follow-up reminder emails were distributed at two-week intervals. It included sections on sociodemographics (six items), the pandemic’s impact on the private sector (27 items), and the public sector (21 items). The instrument was adapted from the validated tool used in 2014 to assess the 2012-2013 economic crisis. Core items assessing patient volume, treatment mix, income changes, and professional stress were retained verbatim to ensure comparability, while COVID-19-specific items (e.g., infection-control protocols, pandemic-related closures) were added without altering the original constructs. The study received ethical approval, and informed consent was obtained.

Results: Responses were received from 145 dentists (14.5% of all registered). Among private practitioners, 39.5% reported fewer patients in 2020 compared with 47.7% in 2012-2013, while the reduction in the public sector was far greater (88.5%, p < 0.001). Income loss was self-reported by 58.9% of private dentists in 2020, compared with 81.3% in the 2014 survey that used the same self-report format. Stress related to reduced income was noted by 62.2% of private dentists (23.5% moderately; 38.7% strongly/very strongly), similar to 71.3% in 2014. By contrast, only 19.2% of public dentists reported stress in 2020, versus 75% in 2014 (p < 0.001). Preventive visits were self-reported as a proportion of total visits and increased to 23.5% in 2020 (vs. 3.9% in 2014), despite an overall reduction in patient volume. By contrast, 95% of public-sector visits continued to be problem-driven.

Conclusion: The COVID-19 pandemic had a marked but uneven impact on dentists in Cyprus. Private dentists experienced significant income loss and stress, whereas salaried public dentists were largely protected. Although less severe than the 2012-2013 crisis, the findings underscore the profession’s vulnerability to external shocks, with consequences for dentists’ well-being and patient oral health.

## Introduction

The COVID-19 pandemic left the health systems of most countries unprepared to deal with situations of such scale and severity. Governments were forced to take tough measures, including lockdowns, to ensure the necessary time to organize health systems, especially in terms of building and equipping intensive care units, purchasing personal protective equipment, and later vaccinating the population [1].

In Cyprus, the first COVID-19 case was registered in March 2020. Coping with the pandemic was clearly a challenge for Cyprus [2], not only because of a lack of experience but also because a new comprehensive National General Healthcare System (GeSY) to ensure universal coverage had just been implemented in 2019, and public hospitals were taking the first steps toward financial and administrative autonomy [3]. Nevertheless, Cyprus demonstrated a commendable response to the COVID‑19 pandemic, as early and decisive non‑pharmaceutical interventions helped prevent healthcare system overload, while its vaccination rollout achieved satisfactory uptake, contributing to effective disease control [4].

Dental practice activity worldwide was significantly affected by the suspension of routine services during the early phases of the pandemic, in accordance with the World Health Organization (WHO) and European Centre for Disease Prevention and Control (ECDC) recommendations to restrict dental care to emergencies only. During the first and second phases of the pandemic, national authorities in several countries, including Cyprus, issued directives for the closure of dental practices, restricting care to emergency cases only. Subsequently, infection control protocols within dental settings were revised and reinforced with more stringent measures, reflecting the high risk of viral transmission inherent to the dental profession. These enhanced requirements increased the use of time and materials and carried important economic consequences because infection-control strategies now had to be balanced against limited resources and feasibility constraints [1].

Currently, there is a dual system of oral healthcare delivery in Cyprus: Dental care is provided by both the Public Dental Services (PDS) and the private sector. The two sectors differ substantially in terms of accessibility and financial resilience: private practitioners depend almost entirely on out-of-pocket payments and are therefore more vulnerable to economic fluctuations, whereas the PDS offers low-cost, state-subsidized services that maintain relatively stable patient flow even during periods of economic stress. The number of practising dentists in 2021 was comparatively high at about 1020 (119 per 100,000 population), but only 3.8% (38) work in the public sector, providing dental care to approximately 10% of the population, mainly people on lower incomes due to the low costs [3,5]. Unfortunately, the new healthcare system that was introduced in 2019 covers only preventive dental care. This limited coverage affects the two sectors differently: while private dentists rely on out-of-pocket payments for the majority of services not reimbursed by GeSY, the PDS continue to provide low-cost, state-subsidized treatment, which buffers them to some extent from fluctuations in patient demand. As a result, most citizens are seeking dental care in the private sector, paying out of pocket. Despite this, during the years 2020-2022, Cyprus recorded lower unmet dental care needs compared with the European Union (EU) average, while in the years 2017-2019, the unmet dental care needs among Cypriots were higher compared with the EU average [6].

In this context, the present study investigates the economic impact of the COVID-19 pandemic on the dental profession in Cyprus, encompassing both the public and private sectors, using a questionnaire based on the instrument employed in the 2014 national study. Core items were retained to allow direct comparison between the two periods. The findings are further compared with data from other countries, as well as with the effects observed during the 2013-2015 financial crisis. In addition, the study explores the psychological burden experienced by dentists during the pandemic.

## Materials and methods

This was a cross-sectional questionnaire-based study conducted among dentists practicing in Cyprus during the coronavirus disease 2019 (COVID-19) pandemic. The primary objective was to assess changes in dental service provision, patient attendance, economic impact, and perceived professional stress associated with the pandemic and the concurrent economic crisis. The study population included licensed dentists working either in the private sector or in the PDS in Cyprus. Both general dentists and dentists providing specialized services were eligible to participate. This inclusive approach was chosen because the study aimed to capture the overall impact of the pandemic on the entire dental workforce in Cyprus and to maintain consistency with the 2014 national survey, which also included both groups. Although general dentists and specialists may experience different operational and financial challenges, both contribute to the national oral healthcare system, and capturing their responses provides a more representative assessment of sector-wide effects. The sampling strategy aimed to reflect the geographical distribution of dentists across the major districts (Nicosia, Limassol, Larnaca-Famagusta, and Paphos), as well as the employment sector (private vs. public). These districts constitute the official administrative divisions of Cyprus and encompass the entire practicing dental workforce; therefore, stratifying the sample by district ensured national representativeness and consistency with the 2014 survey design [7].

An online questionnaire was used, containing 54 questions, and was divided into three parts. Part A contained six questions on demographic characteristics, Part B contained 27 questions on the impact of the pandemic on dentists working in the private sector, and Part C contained 21 questions on the impact of the pandemic on dentists working in the public sector. The questionnaire was designed on the basis of a questionnaire used in a 2014 study by Charalampous et al. [7] to assess the impact of the economic crisis on the dental profession and to allow for comparisons. The original 2014 questionnaire had previously been developed for and used among dentists in Cyprus, thereby establishing its contextual and content validity for this professional group. For the present study, the core items were retained unchanged, and the adapted version was piloted with a small group of Cypriot dentists to ensure clarity, relevance, and suitability for the target population before full distribution.

The term ‘preventive visits’ was defined identically to the 2014 study and referred to routine dental check-ups, scaling and polishing, and recall examinations. Respondents were asked to estimate the proportion of their total patient visits falling into this category during 2020. These data reflect the relative distribution of treatment types, not absolute patient numbers, allowing direct comparison with the 2014 survey. As in the 2014 national survey, income changes were assessed through self-reported categorical items (e.g., increase, no change, decrease), as no objective financial or tax records were collected. To preserve comparability with the 2014 study, all core items assessing changes in patient attendance, provision of specific treatment categories, income impact, and perceived stress were retained verbatim from the original questionnaire. For the COVID-19 context, additional items were included to capture pandemic-specific factors such as mandatory infection-control measures, practice closures, and personal protective equipment usage. These new items were placed in separate subsections and did not modify the wording or structure of the original core items, ensuring that longitudinal comparisons between 2014 and 2022 remained valid. The questionnaires, which included closed questions, were piloted and revised before they were distributed to the potential respondents, together with a letter explaining the nature of the survey and confirming that responses would be confidential and that none of the responders would be identified when the results of the survey were published. The study was approved by the Cyprus National Bioethics Committee (ref. no. 2021.01.178). The questionnaire was administered in Greek through Google Forms (Google LLC, Mountain View, California, USA) and distributed via email to all 1,157 registered dentists in Cyprus (based on the official registry of the Cyprus Dental Association) during April-May 2022. To enhance participation, two follow-up reminder emails were sent at two-week intervals. An online informed consent form was included and required to be completed prior to participation. The links of the Greek and English versions of the questionnaire are shown in the Appendix.

Inclusion criteria were (i) actively practicing clinical dentistry in Cyprus during the data-collection period (April-May 2022), (ii) either self-employed in private practice or employed in the PDS, and (iii) able to complete the questionnaire in full.

Exclusion criteria were (i) dentists not clinically active (e.g., retired, exclusively academic/administrative roles without patient care), (ii) dentists practicing outside Cyprus, and (iii) incomplete questionnaires with missing core outcome items regarding patient volume, income impact, or stress perception. All questionnaires were screened for completeness before analysis. Partially completed forms were excluded if they lacked responses to any of the core outcome items. Only fully completed questionnaires that met all inclusion criteria were retained for the final dataset.

Sampling strategy

A census sampling approach was employed. All 1,157 registered dentists listed in the Cyprus Dental Association registry were invited to participate, regardless of district, sector, or specialty. This method was selected to maximize representativeness and to ensure methodological consistency with the 2014 national survey, which also invited the entire dental workforce.

Sample size and power analysis

At the time of the survey, 1,157 dentists were registered in Cyprus, according to the official registry of the Cyprus Dental Association. A total of 145 dentists completed the questionnaire, representing 14.5% of the total national population of dentists. The adequacy of this sample size was evaluated through a post-hoc power and precision analysis. Using Cochran’s formula for proportions with finite-population correction at a 95% confidence level and a conservative assumption of maximum variability (p = 0.50), the achieved sample provides an FPC-adjusted margin of error of ±7.53%. This precision falls well within the generally accepted range (±5-10%) for national professional surveys.

To assess potential non-response bias, the geographic (district) distribution and private/public sector composition of respondents were compared with the national dentist registry and were found to be broadly similar. Individual demographic data for non-respondents were not available for direct comparison; however, the alignment at the district and sector level suggests that major systematic non-response bias was unlikely.

In addition, to assess statistical power for inferential analyses, a series of two-sided tests (α = 0.05, power = 0.80) was modelled. For a single-proportion test, the minimum detectable difference from a benchmark proportion near 50% was approximately 11% (10.8% when corrected for finite population). For comparisons between two subgroups of similar size (e.g., private vs. public sector), the minimum detectable absolute difference was approximately 23 percentage points [8]. These results indicate that the study had sufficient power to detect moderate-to-large differences and to provide nationally representative prevalence estimates with acceptable precision. Although true random sampling was not feasible in this voluntary, census-based survey design, the post-hoc precision and power calculations were performed under the standard assumption of simple random sampling (design effect = 1) to allow comparison with established survey methodology benchmarks. This analytical assumption does not imply that randomization occurred, but provides a conventional framework for interpreting margin-of-error and power estimates. The sample size methodology followed standard references for survey research and finite-population corrections [8].

Statistical analysis

All data were transferred from Google Forms into Microsoft Excel (Microsoft Corp., Redmond, WA, USA) and analyzed using IBM SPSS Statistics for Windows, version 26.0 (released 2018, IBM Corp., Armonk, NY). Chi-square and Fisher's exact tests were used to compare proportions. The Mann-Whitney or Kruskal-Wallis test was used for the comparison of continuous variables between two or more groups. Spearman correlation coefficients were used to examine the relationship between two continuous variables. All p-values given are two-tailed. Statistical significance was set at p < 0.05.

No formal adjustments were applied for multiple comparisons, as the analyses were primarily exploratory and intended to identify patterns rather than to test a predefined set of hypotheses. Applying strict correction procedures would have increased the likelihood of Type II errors in this context. Therefore, all p-values should be interpreted with caution and in conjunction with effect sizes and clinical relevance.

## Results

The sample size (n) consisted of 145 dentists registered in Cyprus (representing 14.5% of the total number of registered dentists in Cyprus) with a mean age of 47.1 years (standard deviation (SD) = 9.9 years). Individual demographic information for non-respondents (age and gender) was not available; therefore, direct comparison was not possible. However, the geographic distribution (by district) and private/public sector composition of respondents were compared with the national registry and were broadly similar, suggesting no major systematic selection bias in these characteristics. The sample largely mirrored the national dental workforce, showing a predominance of female practitioners and a clear majority working in private practice, while the remainder were employed in the PDS. Of all respondents, 119 (82.1%) were private-sector dentists, and 26 (17.9%) were public-sector dentists. Importantly, although the number of public-sector participants is smaller in absolute terms, it is highly representative: during the study period, a total of 35 dentists were employed in the PDS, meaning that the response rate among public-sector dentists reached 74.2%. Most participants were based in Nicosia, followed by Limassol, Larnaca-Famagusta, and Paphos, closely matching the national geographical distribution. These characteristics and comparisons with both the general population of dentists and the 2014 survey [7] are summarized in Table 1.

**Table 1:** Sample characteristics of participating dentists in 2022, with reference to the national dentist registry and the 2014 survey population. Reference population values are derived from the Cyprus Dental Association registry, and 2014 values are taken from Charalampous et al. (2014) [7] for comparison purposes. These data were not collected in the current survey.

Variable	Category	N (%)	Reference population (%)	Population of dentists participating in 2014 survey (%)
Gender	Men	59 (40.7)	55	46
	Women	86 (59.3)	45	54
Working sector	Private	119 (82.1)	95	84
	Public	26 (17.9)	5	16
District	Nicosia	63 (43.4)	44	40
	Limassol	26 (17.9)	29	28
	Larnaca–Famagusta	14 (9.7)	15	18
	Paphos	16 (11.0)	12	14
Dental services provided	General	88 (73.9)	-	89
	Specialized	31 (26.1)	-	11
Professional experience	Mean years	20.3	-	15.3
Family status	Single	19 (13.1)	-	-
	Married	108 (74.5)	-	-
	Partnership	4 (2.8)	-	-
	Divorced	13 (9.0)	-	-
	Widowed	1 (0.7)	-	-
Number of children	None	23 (15.9)	-	-
	1-2	100 (69.0)	-	-
	3 or more	22 (15.2)	-	-
Country of degree	Greece	93 (64.1)	-	-
	Other EU member state	42 (29.0)	-	-
	Non-EU country	10 (6.9)	-	-

The COVID-19 pandemic markedly disrupted patient attendance patterns. Among private dentists, the number of new patients in 2020 decreased sharply in 37.8% (n = 45) of cases compared to previous years. Absolute patient counts for previous years were not collected; the questionnaire assessed changes relative to prior practice volumes using predefined categorical response options, consistent with the methodology of the 2014 survey. There was also a sharp increase in the number of patients who cancelled a scheduled appointment in 85 (71.4%) participants. In addition, 80 (67.2%), which is more than half of dentists in the private sector, reported an increase in patients asking for payment plans, and 77 (64.7%) patients postponed treatment due to financial difficulties (Table 2). This decline highlights the patients’ shift toward basic and emergency care while delaying elective or aesthetic procedures.

**Table 2:** Changes in the work activity of participating private practice dentists in 2020 compared to previous years.

Activity during 2020 compared to previous years:	Decreased much	Decreased	Remained stable	Increased	Increased much	Do not offer this service
	Ν (%)	Ν (%)	Ν (%)	Ν (%)	Ν (%)	Ν (%)
No. of new patients	7 (5.9)	38 (31.9)	41 (34.5)	32 (26.9)	1 (0.8)	-
Patients who canceled scheduled appointment	5 (4.2)	10 (8.4)	19 (16)	60 (50.4)	25 (21)	-
Patients who did not pay part of or whole therapy	3 (2.5)	5 (4.2)	68 (57.1)	36 (30.3)	7 (5.9)	-
Patients who asked for a discount	4 (3.4)	1 (0.8)	52 (43.7)	51 (42.9)	11 (9.2)	-
Patients who asked for special paying arrangements	2 (1.7)	2 (1.7)	35 (29.4)	62 (52.1)	18 (15.1)	-
Patients who postponed therapy due to financial difficulties	1 (0.8)	6 (5)	35 (29.4)	65 (54.6)	12 (10.1)	-
Number of fixed prosthetics	14 (11.8)	33 (27.7)	31 (26.1)	16 (13.4)	2 (1.7)	23 (19.3)
Number of implants used	14 (11.8)	31 (26.1)	28 (23.5)	14 (11.8)	3 (2.5)	29 (24.4)
Number of orthodontic appliances	16 (13.4)	15 (12.6)	25 (21)	13 (10.9)	0 (0)	50 (42)
Income	5 (4.2) (> 50%)	64 (54.7)	34 (28.6)	15 (12.6)	0 (0.0) (>50%)	-

Absolute numbers of patient visits in previous years were not collected; the questionnaire assessed changes relative to prior attendance using predefined categorical options (e.g., increased, decreased slightly, decreased sharply), consistent with the methodology of the 2014 survey. Therefore, the comparisons reported reflect relative shifts rather than exact visit counts.

The reduction in the provision of expensive dental treatments (e.g., prosthetics, implants and orthodontics) was also significant: 47 (48.9%) of the 96 participating private practice dentists who offered this treatment stated that there was a decrease or great decrease in the construction of fixed prosthetic work, 45 (50%) out of 90 in implant placements and 31 (44.9%) out of 69 in orthodontic treatments (Table 2).

Reduced daily patient numbers were significantly more common among private-sector dentists than their public-sector counterparts. Among private practice dentists, 47 (39.5%) reported that their daily number of patients had fallen or fallen sharply. A significant difference was also observed between the private and public sectors, as 23 (88.5%) of public sector dentists reported decreases (p < 0.001) (Table 3).

**Table 3:** Comparison of working characteristics between private and public sector. Data are shown as mean ± standard deviation (SD) or number (N (%)), as appropriate. Statistical tests: Mann–Whitney U test (+), Pearson’s Chi-square (++), and Fisher’s exact (‡). A p < 0.05 was considered statistically significant.

		Private sector	Public sector	
		N (%)	N (%)	P
Daily number of patients during 2020 compared to previous years:				
	Much decreased	9 (7.6)	4 (15.4)	<0.001+
	Decreased	38 (31.9)	19 (73.1)	-
	Stable	47 (39.5)	2 (7.7)	-
	Increased	24 (20.2)	1 (3.8)	-
	Much increased	1 (0.8)	0 (0)	-
Reason for patients’ visit				
	Prevention/ Regular check	28 (23.5)	1 (3.8)	0.023++
	Dealing with a problem	91 (76.5)	25 (96.2)	-
Has the reason for patients’ visit changed compared to previous years?				
	No	66 (55.5)	21 (80.8)	0.017++
	Yes	53 (44.5)	5 (19.2)	-
Feeling stressed about the consequences of the pandemic on your income?				
	Not at all	8 (6.7)	10 (38.5)	<0.001+
	A little	37 (31.1)	11 (42.3)	-
	Moderately	28 (23.5)	3 (11.5)	-
	Much	38 (31.9)	2 (7.7)	-
	Very much	8 (6.7)	0 (0)	-
The new working condition due to the pandemic cause you:				
	More stress than before	91 (76.5)	18 (69.2)	0.129‡
	Same stress as before	27 (22.7)	6 (23.1)	-
	Less stress than before	1 (0.8)	2 (7.7)	-
+Mann-Whitney test; ++Pearson's chi-square test; ‡Fisher's exact test				

Income loss followed a similar pattern. Regarding the impact of the pandemic on income, 70 (59.3%) of private sector dentists reported that their income had decreased/severely decreased in 2020 (Table 2). In addition, 28 (23.5%) stated that they felt moderate stress, and 46 (38.7%) felt a great deal or very much of stress because of the impact of the pandemic on their income. Public-sector dentists felt significantly less stressed about the impact of the pandemic on their income (p < 0.001). Overall, 38.5% reported no stress, 42.3% reported a little stress, 11.5% reported moderate stress, and only 7.7% reported much stress, with none reporting very high stress (Table 3).

Patients’ reasons for visiting the dentist also shifted. Preventive or routine check-ups were more commonly reported among private dentists (76.5%) than among those in the public sector (69.2%), whereas emergency-only visits increased markedly in both groups. Moreover, private dentists were more likely to note that patients’ reasons for attendance had changed compared with previous years, reinforcing the economic and behavioural dimensions of the pandemic’s impact (Table 3). These proportions refer to the relative distribution of visit types within 2020 only and do not contradict earlier findings regarding a reduction in preventive care compared with previous years. As defined in the questionnaire (and consistent with the 2014 methodology), “preventive visits” included routine check-ups, scaling and polishing, and recall examinations. The observed sectoral differences, therefore, reflect the composition of visits during 2020, not changes relative to the pre-pandemic period.

Satisfaction with governmental support during lockdown remained low among private practitioners, with an average score of 2.7 on a 1-5 scale, underscoring a perceived gap between policy measures and the profession’s actual needs.

Within the private sector, lower perceived stress was associated with greater satisfaction regarding government financial support measures and with reports of improved or stable income (in relation to the period March-June 2020). Conversely, dentists who felt more anxious while providing care during the pandemic also reported higher stress levels related to financial strain (in relation to the period November 2020-January 2021). These relationships remained significant in the multivariable regression model presented in Table 4, which examined predictors of income-related anxiety among private dentists. In this model, perceived income reduction, lower satisfaction with government financial-support measures, and greater clinical anxiety during the pandemic emerged as the strongest independent predictors of income anxiety.

**Table 4:** Results of multiple linear regression results on private dentists' income anxiety as the dependent variable. Data are presented as regression coefficients (β) with standard errors (SE) and p-values. Professional experience measured in years. A p < 0.05 was considered statistically significant.

	β+	SE++	P
Gender (women vs. men)	0.064	0.033	0.056
Age	-0.004	0.007	0.537
Additional degree beyond bachelor's in dentistry (yes vs. no)	-0.024	0.039	0.540
Married/civil union/partnership (yes vs. no)	-0.014	0.039	0.725
Years of practice	0.002	0.006	0.713
Your professional income in 2020 compared to before	-0.039	0.015	0.011
Financial measures to support dental practices during the lockdown were satisfactory in the period March-June 2020	-0.035	0.014	0.012
You felt more anxiety while providing dental care in the period November 2020-January 2021	0.030	0.015	0.045

## Discussion

The sociodemographic characteristics of the sample are consistent with the reference population, but also with the characteristics of the 2014 survey sample [5,7], which investigated the impact of the financial crisis on the dental profession, allowing for a reliable comparison.

The results of the survey showed that the pandemic had a negative economic impact on the dental profession and generated considerable stress and uncertainty among dentists. Importantly, the magnitude and nature of this impact varied markedly between sectors: private-practice dentists were disproportionately affected financially and reported higher levels of stress, whereas public-sector dentists experienced fewer income disruptions and different operational challenges.

The decrease in daily patient numbers and in the number of new patients in 2020 compared to previous years was due to many pandemic-related reasons, including restrictive operational measures, the longer time dentists needed to provide dental care to their patients in order to implement the new infection control protocols [1,9,10], and also to patients' fears and hesitation or reluctance to visit the dentist [11]. Information campaigns encouraging citizens to limit social contact and avoid unnecessary travel also reduced dental visits [12]. Consequently, the restrictive measures that were taken, and which affected the operations of businesses and the economy in general, also deterred patients from seeking dental care [13], especially in the private sector, where patients bear the costs themselves (self-paying), as the Cypriot health system only reimburses preventive dental care. It is well known and documented that dental care has the highest financial barriers for patients compared to other health sectors [14].

In the present study, 39.5% of the private practice dentists reported a decrease in the daily number of patients, and 71.4% reported that there was an increase in the number of patients who cancelled scheduled appointments, which is consistent with other studies [15-17]. However, in the literature, there is a variation in the extent of the reported reduction in the daily number of patients. This discrepancy could be due to the different number of COVID-19 cases recorded in each country, as well as the lack of clear universal regulations for the operation of dental practices in the different countries, which in turn affected patient choice.

The percentage of dentists in the public sector who reported that their daily patient load had decreased was significantly higher (88.5% vs. 39.5% p < 0.001), a fact that may be related to the different profile of patients in the public sector (older patients who belonged to vulnerable populations that were more restricted during the pandemic) on the one hand, and the obligation to follow Cyprus Ministry of Health regulations and limit the scope of their work to emergency care on the other. It is clear that dentists in the public sector receive a salary, and the decline in patient numbers does not affect their income, unlike private practice dentists. In addition, dental staff working in the PDS were diverted to tasks related to COVID-19 management, such as tracking COVID-19 positive cases. The great decrease in the number of patients visiting the public sector during the pandemic is also confirmed by the statistics of the PDS [18].

Accordingly, in a study conducted in Turkey, respondents working in private practices cared for the most patients, and respondents working in public institutions cared for the least number of patients [19]. By contrast, in central Italy, mainly only emergency treatments were carried out during the lockdown, showing that most professionals followed the advice of the authorities [20]. In Spain, another country with an increased number of COVID-19 cases, 25.7% of dentists communicated with their patients by phone in April 2020, while 25% stopped working completely [21].

One possible interpretation is that variation in how restrictive measures were implemented across private practices may have influenced patient flow during this period; however, the present study did not directly assess compliance, and this explanation should be considered cautiously. This is also supported by the fact that dentists' satisfaction with the support measures offered by the government to dental practices while the restrictive measures were in place was low (2.72 on a scale of 1-5). A similarly low level of satisfaction (2.41, SD = 0.924) was also expressed by dentists in Turkey [19]. It becomes clear that if the state has to take such drastic measures as closing dental practices, it should consider the wider socio-economic impact and be prepared to take appropriate countermeasures.

An older age and more years in the profession of private practice dentists were positively related to a greater likelihood of reporting a decrease in the number of daily patients. Several contextual factors may help explain this pattern. Older dentists may have had greater financial stability or may have reduced clinical activity to minimise personal risk, whereas younger practitioners might have been more flexible in pricing or appointment availability. These possibilities are interpretive and should be viewed cautiously, as the survey did not directly measure motivations or behavioural responses.

As far as the economic consequences of the pandemic were concerned, 58.9% of private dentists reported that their income had decreased in 2020 compared to previous years, threatening the economic viability of the profession. The high density of dentists in Cyprus, reported to be among the highest in Europe [22], may also contribute to increased competition within the profession and could partly explain the vulnerability of private-practice income. This interpretation is consistent with general economic principles relating provider density to income levels, although the present study did not directly assess this relationship. In addition, nearly two in three private practice dentists (64.7%) reported an increase in the number of their patients who postponed dental treatment due to financial difficulties. Of course, this percentage was lower than that found by the 2014 survey, where 90.7% of private practice dentists answered positively to the same question [5]. In a study that was conducted to investigate the operating conditions of dentists in Central Europe during the first COVID-19 lockdown, almost three-quarters of Swiss dentists reported an income drop by 80-100% in the most unprofitable months [23].

As expected, a notable proportion of dentists reported reductions in high-cost procedures, including fixed prosthetics (49.8%), implants (50%), and orthodontics (44.9%). These percentages represent descriptive frequencies from our survey; no statistical comparison was performed between these procedure categories. These percentages are still lower than those during the financial crisis, when the corresponding percentages were 70.6%, 73.4% and 53.1% respectively. Of course, the decrease in demand for the specific treatments is not only related to their cost, but also to its nature, because, on the one hand, the prosthetic appliances and implants require the use of an air rotor and therefore the production of aerosols and consequently a greater risk of transmission of the virus, and, on the other hand, they are not considered urgent dental procedures. In Poland, 60.2% of the responding dentists mentioned that during the first wave of the pandemic, they did not perform crown and bridge work [20]. The recommendations of the World Health Organization (WHO) and other health organizations have resulted in a decrease in the demand for aesthetic dental treatment. Although there are many and highly variable results on this topic in the literature [10], in general, lockdowns and closures of dental practices have had a significant impact on dentists' income [23].

As a result of the decline in patient numbers and dentists’ income, 38.6% of private dentists reported that they felt much or very much stressed by the impact of the pandemic on their income. Jungo et al. [24] reported that dentists in France were more concerned about the financial viability of their practice than about contracting the virus. Financial hardship related to concerns about loss of job or income, as well as unexpected investment in infection control procedures, was cited as a trigger for financial stress [25]. The perception of the pandemic as a financial hazard is one of the important factors in increasing the level of distress [26, 27]. Less stress was reported by private practice dentists who reported that their income was not affected during the pandemic, and those who reported that government support measures for the dental profession were satisfactory. The percentage of practising dentists who reported feeling stressed by the impact of the pandemic on their income was lower compared to the equivalent of the 2014 financial crisis (44.9%), suggesting that the pandemic, as a different form of crisis, was less intense and extensive than the financial crisis. A much lower percentage of dentists in the public sector reported feeling stressed by the impact of the pandemic on their income (7.7% p < 0.001), a difference that was to be expected as dentists in the public sector are on the government payroll, and in no case did the issue of a salary cut arise during the pandemic. On the contrary, during the period of economic crisis, the percentage of public sector dentists who reported feeling stressed by the impact of the economic crisis on their income was correspondingly high (37.5%), as the crisis was accompanied by salary cuts.

This sectoral contrast highlights an important dimension of policy resilience during health emergencies: salaried employment within state-funded systems appears to buffer clinicians from income-related stressors, protecting professional stability even when patient numbers decline substantially. In contrast, private practitioners, whose income depends directly on patient flow, remain more vulnerable to economic shocks, underscoring the need for targeted financial-support mechanisms for self-employed health professionals during future crises.

Another and maybe more important source of stress for the dentists, except from the financial hardship, was the new and more demanding working conditions. The increased risk of virus transmission in dental work and the need to adopt stricter protocols [1, 26], as well as the obligation of finding and properly using personal protective equipment and closely following instructions on disinfection and sterilisation, were important stressors for dentists in both the public and private sectors (76.5% and 69.2%, respectively, percentages that were higher compared with those that were recorded among Cypriots’ dentists regarding the stress they felt by the impact of the pandemic on their income). Anxiety and fear of COVID-19 transmission among dentists during dental procedures were also found in other countries, such as India [17]. Ahmed et al. [28], in their study among dentists from 30 different countries, found that fear of their family members’ being infected, fear, and economic anxiety were among the psychological effects of the COVID-19 pandemic. It is interesting to note that in a survey in Colombia, 26.5% of dentists said they were thinking of retiring earlier due to the pandemic, 18.1% to changing their profession, and 16% to following a non-clinical dental activity [29]. By contrast, in Australia, a lower percentage of dentists (28.9%) reported feeling moderately or extremely stressed at work due to the COVID-19 outbreak, which is probably related to the overall low number of COVID-19 cases and minimal community transmission in Australia [30].

The pandemic also affected the reasons why patients sought dental care, with notable differences between the public and private sectors. Almost all dentists in the public sector reported that their patients attended primarily because of a problem, compared with 76.5% in the private sector (p < 0.05). This disparity likely reflects differences in the socioeconomic profile and insurance coverage of patients utilizing each system. Patients relying on the PDS often belong to lower-income or socially vulnerable groups and may be less likely to seek preventive care unless symptomatic, even though fees are very low. Additional factors may include limited awareness of the importance of routine preventive visits and structural elements of the public system, such as the absence of productivity-linked incentives for encouraging regular recall appointments. These parameters together may help explain the predominance of problem-driven visits observed among public-sector patients.

In addition, limited working hours of the PDS and lack of time among PDS’s patients are among the factors that reduce the likelihood of visiting the PDS. It should be noted that during the financial crisis, 95% of dentists in both the public and private sectors reported that their patients were seeing them for a dental problem. This situation seems to have improved later in the private sector, which is why a significantly higher percentage of private practice dentists reported that the reason for their patients' visits has changed compared to before. The decrease in preventive visits may have an impact on the level of oral health of the citizens of Cyprus in the medium and long term, as preventive dental visits are associated with a reduction in treatment needs [31]. Although longitudinal data for Cyprus are not yet available, this trend highlights the importance of future follow-up studies to monitor whether the decline in preventive attendance translates into increased treatment needs or worsening oral health outcomes over time. Lower demand for preventive dentistry was also found in the US during September 2019- December 2020 [13]. In a survey conducted in the Czech Republic, 47.3% of dentists stated that they had noticed a decrease in their patients' interest in preventive care during the pandemic. The same survey also found that 16.9% of dentists reported that their patients were less interested in their oral health, a fact that the authors believe may contribute significantly to the decline in overall oral health in the Czech Republic [15].

Strengths and limitations

A major strength of this study is that it represents the first national attempt to directly compare the consequences of the COVID-19 pandemic with those of the economic crisis, using the same validated research tool. This unique feature adds a layer of interest and robustness to the findings. It should be noted that the response rate was calculated using the total number of registered dentists in Cyprus as the denominator, as official data distinguishing actively practicing dentists was not available. Because not all registered dentists necessarily practice clinically, the effective response rate among eligible, actively practicing dentists is likely higher than the nominal 14.5% reported.

At the same time, several limitations merit consideration.

Because the survey was conducted in 2022 and asked dentists to report experiences from 2020-2021, recall bias is possible and may have influenced the accuracy of retrospective estimations of patient attendance, income changes, and stress levels.

The overall response rate, while sufficient for statistical analysis, was modest. This may introduce non-response bias, particularly if dentists experiencing greater financial hardship or stress were either more or less motivated to participate. As data on non-respondents were unavailable, the direction and magnitude of this potential bias cannot be empirically verified, and the findings should therefore be interpreted with appropriate caution. A lack of detailed contextual variables, such as whether dentists practiced individually or in group settings, or the relative size of their patient base, also restricted deeper interpretation of the findings. Nevertheless, the data provide valuable insights into differences between public and private practitioners regarding income, workload, and professional well-being.

It is worth underlining that relatively low participation rates are a common phenomenon in surveys addressing the entire dental workforce of a country, given the heavy clinical workload, the large number of concurrent surveys, and, at times, a reduced level of engagement or awareness among colleagues. In addition, online distribution via email may have contributed to the modest response rate, as email surveys are susceptible to non-engagement, spam filtering, and survey fatigue. Although administering the questionnaire physically might improve participation, this was not feasible during the COVID-19 period due to infection-control restrictions and the need to minimize face-to-face contact.

Comparisons with the 2014 survey [10] should also be approached with some caution. While both surveys highlight similar challenges, the healthcare environment has since changed considerably with the implementation of the General Healthcare System (GeSY). Its preventive orientation and influence on access to dental services represent factors that were not present in 2014 [7], and which may partially account for differences between the two survey periods.

Despite these considerations, the study retains strong value. It demonstrates clear and consistent trends regarding reductions in income and activity levels, as well as heightened professional anxiety, across both private and public dentists. Although the relatively modest sample size advises careful interpretation of statistical significance, the findings remain indicative of important shifts within the dental profession in Cyprus and provide a useful basis for future research and policy planning.

## Conclusions

The findings of this study indicate that the COVID-19 pandemic was associated with reduced patient attendance in dental practices in Cyprus, particularly within the private sector, and that these changes coincided with reported financial strain and heightened professional stress among dentists. Although the overall impact appeared less severe than that experienced during the 2014 financial crisis, the results suggest that the dental profession may remain sensitive to external disruptions. For context, 81.3% of private dentists reported income loss during the 2014 financial crisis, compared with 58.9% in 2020, reinforcing that the economic impact of the pandemic, while substantial, was less severe than that of the earlier crisis. This sensitivity may have implications not only for practitioners but also for patients, especially if reduced attendance limits opportunities for preventive care.

Future longitudinal or follow-up studies are warranted to determine whether these reductions in preventive attendance translate into measurable changes in oral-disease burden and treatment needs in the Cypriot population. While these patterns cannot establish causality, they highlight areas where strengthening the resilience and preparedness of dental services could help mitigate disruptions during future public health emergencies. Potential strategies could include targeted financial-support mechanisms for private practitioners during periods of restricted clinical activity, clearer national contingency protocols for dental services, and the integration of oral healthcare into broader emergency preparedness planning. Given the unique structure of the Cypriot dental system and the timing of national restrictions, these findings are context-specific, and caution is warranted when considering their generalizability beyond Cyprus.

## Appendices

English version of the questionnaire: https://pdflink.to/afc857de/

Greek version: https://pdflink.to/00db7f7f/

## References

[1] Volgenant CM, Persoon IF, de Ruijter RA, de Soet JJ (2021). Infection control in dental health care during and after the SARS-CoV-2 outbreak. Oral Dis.

[2] Gountas I, Quattrocchi A, Mamais I, Tsioutis C, Christaki E, Fokianos K, Nikolopoulos G (2021). Effect of public health interventions during the first epidemic wave of COVID-19 in Cyprus: a modelling study. BMC Public Health.

[3] Theodorou M, Charalambous C, Williams GA (2024). Cyprus: health system review. Health Syst Transit.

[4] Giannakou K, Fakonti G, Kyprianidou M (2022). Determinants of COVID-19 vaccine uptake among healthcare professionals and the general population in Cyprus: a web-based cross-sectional survey. J Eval Clin Pract.

[5] Charalambous C, Theodorou M, Eaton KA (2020). The system of the provision of oral healthcare in the Republic of Cyprus and the effect of the economic crisis. Oral Health Prev Dent.

[6] Eurostat Eurostat (2025). Self-reported unmet needs for dental examination by sex, age, main reason declared and degree of urbanisation. Eurostat.

[7] Charalambous C (2017). Epidemiological study evaluating the level of oral health among citizens of Cyprus and the impact of the economic crisis. Open University of Cyprus.

[8] Cochran W (1977). Sampling techniques third edition. https://educationexclusive.com/upload/pdf/Sampling%20Techniques-William%20G.%20Cochran.pdf.

[9] (2025). PIO (Press Information Office Cyprus): coronavirus. https://www.gov.cy/pio/en/.

[10] Sozkes S, Olszewska-Czyż I (2021). Effects of COVID-19 pandemic on working conditions of dentists in Poland and Turkey. Medicina (Kaunas).

[11] Nikolić M, Mitić A, Petrović J, Dimitrijević D, Popović J, Barac R, Todorović A (2022). COVID-19: another cause of dental anxiety?. Med Sci Monit.

[12] Coulthard P, Thomson P, Dave M, Coulthard FP, Seoudi N, Hill M (2020). The COVID-19 pandemic and dentistry: the clinical, legal and economic consequences - part 1: clinical. Br Dent J.

[13] Choi SE, Mo E, Sima C (2024). Impact of COVID-19 on dental care utilization and oral health conditions in the United States. JDR Clin Trans Res.

[14] Brian Z, Weintraub JA (2020). Oral health and COVID-19: increasing the need for prevention and access. Prev Chronic Dis.

[15] Schmidt J, Waldova E, Balkova S, Suchanek J, Smucler R (2021). Impact of COVID-19 on Czech dentistry: a nationwide cross-sectional preliminary study among dentists in the Czech Republic. Int J Environ Res Public Health.

[16] Wolf TG, Deschner J, Schrader H, Bührens P, Kaps-Richter G, Cagetti MG, Campus G (2021). Dental workload reduction during first SARS-CoV-2/COVID-19 lockdown in Germany: a cross-sectional survey. Int J Environ Res Public Health.

[17] Shivani K, Daisy H (2022). Impact of COVID-19 pandemic on dental practice: a questionnaire survey. J Clin Diagn Res.

[18] Charalambous C, Syngelakis AI, Kantaris M (2023). Impact of the COVID-19 pandemic on the provision of dental care by the public dental services of Cyprus. Eur J Dent Oral Health.

[19] Çelik OE, Cansever İH (2021). Evaluation of the effects of the COVID-19 pandemic on dentistry. Clin Exp Dent Res.

[20] Sinjari B, Rexhepi I, Santilli M, D Addazio G, Chiacchiaretta P, Di Carlo P, Caputi S (2020). The impact of COVID-19 related lockdown on dental practice in central italy-outcomes of a survey. Int J Environ Res Public Health.

[21] Martínez-Beneyto Y, Ausina-Márquez V, Expósito-Delgado AJ, Ortiz-Ruiz AJ, Ibañez-Lopez FJ, Llodra-Calvo JC, Bravo M (2021). Spanish dentists' awareness, knowledge, and practice regarding COVID-19: a multiple regression analysis. Int Dent J.

[22] Eurostat Eurostat, 2020 2020 (2025). Healthcare personnel statistics - dentists, pharmacists and physiotherapists - Statistics Explained. https://ec.europa.eu/eurostat/statistics-explained/index.php?title=Healthcare_personnel_statistics_-_dentists,_pharmacists_and_physiotherapists.

[23] Wolf TG, Zeyer O, Campus G (2020). COVID-19 in Switzerland and Liechtenstein: a cross-sectional survey among dentists' awareness, protective measures and economic effects. Int J Environ Res Public Health.

[24] Jungo S, Moreau N, Mazevet ME, Ejeil AL, Biosse Duplan M, Salmon B, Smail-Faugeron V (2021). Prevalence and risk indicators of first-wave COVID-19 among oral health-care workers: a French epidemiological survey. PLoS One.

[25] Mishra S, Singh S, Tiwari V, Vanza B, Khare N, Bharadwaj P (2020). Assessment of level of perceived stress and sources of stress among dental professionals before and during the COVID -19 outbreak. J Int Soc Prev Community Dent.

[26] Barabari P, Moharamzadeh K (2020). Novel coronavirus (COVID-19) and dentistry-a comprehensive review of literature. Dent J (Basel).

[27] Mekhemar M, Attia S, Dörfer C, Conrad J (2021). The psychological impact of the COVID-19 pandemic on dentists in Germany. J Clin Med.

[28] Ahmed MA, Jouhar R, Ahmed N, Adnan S, Aftab M, Zafar MS, Khurshid Z (2020). Fear and practice modifications among dentists to combat novel coronavirus disease (COVID-19) outbreak. Int J Environ Res Public Health.

[29] Plaza-Ruíz SP, Barbosa-Liz DM, Agudelo-Suárez AA (2021). Impact of COVID-19 on the knowledge and attitudes of dentists toward teledentistry. JDR Clin Trans Res.

[30] Sotomayor-Castillo C, Li C, Kaufman-Francis K (2022). Australian dentists' knowledge, preparedness, and experiences during the COVID-19 pandemic. Infect Dis Health.

[31] Taylor HL, Sen B, Holmes AM, Schleyer T, Menachemi N, Blackburn J (2022). Does preventive dental care reduce nonpreventive dental visits and expenditures among Medicaid-enrolled adults?. Health Serv Res.

